# Peptide Hormones in Appetite Regulation: A Complex Network

**DOI:** 10.3390/ph19070989

**Published:** 2026-06-25

**Authors:** Sara Abdollahi, Hussan Adam, Othman Al Musaimi

**Affiliations:** 1 School of Pharmacy, Newcastle University, Newcastle upon Tyne NE1 7RU, UK; s.abdollahi2@newcastle.ac.uk (S.A.); h.adam2@newcastle.ac.uk (H.A.); 2 Translational and Clinical Research Institute, Faculty of Medical Sciences, Newcastle University, Newcastle upon Tyne NE2 4HH, UK; 3 Department of Chemical Engineering, Imperial College London, London SW7 2AZ, UK; 4 Orthogonal Peptides Limited, London SW7 2AZ, UK

**Keywords:** appetite regulation, gut–brain axis, GLP-1, ghrelin, leptin, obesity, hypothalamus, neuroendocrine signalling, peptide YY, energy homeostasis

## Abstract

**Background**: Appetite regulation is governed by a complex neuroendocrine network that integrates peripheral peptide signals with hypothalamic and brainstem circuits to coordinate energy intake and maintain energy homeostasis. Disruption of these pathways contributes to obesity and other disorders characterised by dysregulated feeding behaviour. **Objective**: To map and synthesise the current evidence on the role of appetite-regulating peptide hormones and central neural pathways in appetite control, obesity pathophysiology, and emerging therapeutic approaches. **Methods**: A scoping review of the literature was conducted to identify and synthesise evidence relating to the physiological and pathological mechanisms of appetite regulation. The review examined the actions of key peptide hormones, including ghrelin, glucagon-like peptide-1 (GLP-1), peptide YY (PYY), leptin, and insulin, their interactions within the gut–brain axis, and their effects on central appetite-regulating circuits. **Results**: The evidence highlights the central role of the arcuate nucleus in integrating peripheral hormonal signals with neural pathways controlling feeding behaviour. Appetite regulation is mediated by the balance between orexigenic neuropeptide Y/agouti-related peptide (NPY/AgRP) neurons and anorexigenic pro-opiomelanocortin/cocaine- and amphetamine-regulated transcript (POMC/CART) neurons, with further modulation by the paraventricular, lateral, and ventromedial hypothalamic nuclei. The literature identifies hormone resistance, impaired satiety signalling, and altered neuroendocrine feedback as major contributors to obesity. Evidence on therapeutic interventions demonstrates the potential of GLP-1 receptor agonists, including liraglutide and semaglutide, and the dual incretin agonist tirzepatide, while also highlighting challenges related to treatment durability, adverse effects, and weight regain following discontinuation. **Conclusions**: Current evidence demonstrates that appetite regulation involves highly interconnected peripheral and central signalling pathways. The reviewed literature supports the development of multi-target and precision-based therapeutic strategies for obesity and identifies important areas for future research, including mechanisms of treatment resistance, long-term efficacy, and inter-individual variability in neuroendocrine responses.

## 1. Introduction

Homeostasis is a fundamental physiological process that preserves internal stability and dynamic equilibrium in response to changing external conditions [1]. It regulates essential parameters, including metabolism, pH, temperature, oxygen levels, and blood glucose, thereby maintaining optimal conditions for cellular activity and enzymatic function [1]. This regulation is achieved through both local mechanisms, such as paracrine and autocrine signalling, and systemic reflex pathways involving coordinated interactions between the nervous and endocrine systems [2].

Appetite represents the physiological drive to consume food and is controlled by a complex, integrative network that processes signals from the external environment, gastrointestinal tract (GI), adipose tissue, and central nervous system [3]. For this review, appetite regulation is considered across two interconnected levels: central regulatory system (CRS) and the peripheral system (PS). The CRS includes key structures such as the hypothalamus, particularly the arcuate nucleus (ARC), the nucleus tractus solitarius (NTS) in the brainstem, and the vagus nerve. These centres integrate hormonal and neural inputs from peripheral tissues to regulate sensations of hunger and satiety. Together, these mechanisms ensure the maintenance of energy balance, body weight, and overall homeostasis [4].

Peptides are short chains of amino acids, typically comprising between two and fifty residues linked by covalent peptide bonds formed through condensation reactions. In the human body, peptides perform essential roles in a wide range of physiological processes. Many function as signalling molecules, transmitting information between the brain and peripheral organs to regulate key functions such as metabolism, appetite, and hormone secretion [5]. They are also central to intercellular communication, mediating interactions between peripheral tissues and the central nervous system through specific protein and receptor binding [6].

Peptide function is closely determined by molecular structure, including amino acid composition, sequence length, and secondary conformation. Structural elements such as α-helices, β-sheets, and peptide bond orientation influence receptor specificity, biological stability, and signalling efficiency [7,8]. These relationships underpin the structure–activity relationship (SAR), whereby even minor modifications in amino acid sequence or backbone configuration can result in significant changes in biological activity, receptor affinity, and resistance to enzymatic degradation [9]. Comparative studies of conventional α-peptides and synthetic β-peptides illustrate the importance of backbone structure in determining peptide behaviour. α-peptides, composed of standard amino acids, predominate in physiological signalling systems, such as GLP-1. In contrast, β-peptides contain an additional carbon in the backbone, altering their folding patterns and enhancing resistance to enzymatic degradation. These structural differences influence receptor interactions, selectivity, pharmacokinetics, and overall therapeutic potential [10].

Bioactive peptides represent a diverse group of functional molecules derived from endogenous protein processing, dietary sources, or chemical synthesis. They exert their effects through multiple mechanisms: many bind to cell surface receptors as agonists or antagonists, while others can cross cell membranes to act intracellularly [11]. Their amino acid sequence, stability, and physicochemical properties collectively determine their bioavailability and functional outcomes [12]. Increasing evidence highlights their roles in metabolic regulation, immune modulation, and neuroendocrine signalling, underscoring their growing therapeutic relevance in metabolic disorders [9]. Despite their potential, the clinical application of many peptides is limited by rapid enzymatic degradation and challenges in delivery. Consequently, modern therapeutic strategies increasingly rely on peptide engineering and advanced formulation approaches to enhance stability, prolong half-life, and improve bioavailability [13,14].

Neuropeptides are a specialised class of bioactive peptides synthesised and released by neurons to regulate neuronal communication, endocrine function, and behaviour. Unlike classical neurotransmitters, which act rapidly at synapses, neuropeptides typically produce slower but more sustained effects through activation of G protein-coupled receptors (GPCRs), enabling fine modulation of neural circuits involved in homeostasis and motivation [15]. In the regulation of energy balance, neuropeptides play a central role in integrating peripheral metabolic signals with central neural networks. Orexigenic neuropeptides, such as neuropeptide Y (NPY) and agouti-related peptide (AgRP), stimulate appetite, whereas anorexigenic neuropeptides, including pro-opiomelanocortin (POMC)-derived peptides such as α-melanocyte-stimulating hormone (α-MSH), and cocaine- and amphetamine-regulated transcript (CART), act to suppress food intake. Many of these appetite-regulating peptides are expressed in the hypothalamus, particularly within the ARC, where they respond to circulating hormonal signals such as insulin, leptin, and ghrelin [16]. Through this integration, neuropeptides coordinate feeding behaviour, satiety, and energy expenditure, serving as critical mediators between the gut, adipose tissue, pancreas, and the brain [17]. These characteristics make neuropeptide systems highly suited to the regulation of appetite and highlight their potential as therapeutic targets for metabolic disorders.

Given the breadth of evidence spanning peptide structure, hypothalamic circuitry, peripheral hormone signalling, gut–brain communication, microbiota interactions, and pharmacological modulation, a scoping review approach was used to map the field and identify major mechanistic and therapeutic themes. This scoping review aims to map current evidence on peptide-mediated appetite regulation across central and peripheral pathways, identify key regulatory mechanisms within the gut–brain axis, and summarise emerging therapeutic strategies targeting these systems in metabolic disease.

## 2. Methods

### 2.1. Protocol and Reporting Framework

This scoping review was conducted to map the available evidence on peptide hormones involved in appetite regulation, with particular focus on central and peripheral signalling pathways, gut–brain communication, microbiota-related mechanisms, appetite dysregulation, and therapeutic modulation. The review was reported in accordance with the Preferred Reporting Items for Systematic reviews and Meta-Analyses extension for Scoping Reviews (PRISMA-ScR), where applicable. The aim was to identify, organise, and synthesise the breadth of evidence in this field rather than to perform a quantitative meta-analysis [18].

### 2.2. Search Strategy and Information Sources

Literature searches were conducted between September and December 2025 using PubMed/National Library of Medicine, Web of Science, ScienceDirect, SciFinder, and Google Scholar. ScienceDirect was used to access Elsevier-hosted journal articles. Google Scholar was used as a supplementary source to identify relevant articles not captured in database searches; for each major search combination, the first five pages of results were screened because results beyond this point were considered less relevant to the review aim. Reference lists of relevant review articles and key primary studies were also manually screened to identify additional sources.

Search terms included combinations of the following: appetite regulation, satiety, hunger, energy homeostasis, peptide hormones, neuropeptides, hypothalamic appetite regulation, gut–brain axis, vagal afferent signalling, gut microbiota, ghrelin, leptin, insulin, PYY, GLP-1, GIP, oxyntomodulin, amylin, cholecystokinin, AgRP, NPY, POMC, α-MSH, obesity, leptin resistance, binge eating disorder, anorexia nervosa, GLP-1 receptor agonists, tirzepatide, semaglutide, cagrilintide, multi-agonist peptides, and anti-obesity pharmacotherapy.

The search prioritised recent evidence, particularly studies published between 2020 and 2026. Earlier foundational studies were included where they provided essential background on peptide discovery, receptor signalling, hypothalamic appetite circuits, or landmark therapeutic development.

### 2.3. Eligibility Criteria

Articles were included if they were published in English and directly relevant to appetite regulation, peptide hormones, neuroendocrine signalling, gut–brain communication, microbiota-related appetite mechanisms, appetite dysregulation, obesity, or peptide-based therapeutic strategies. Eligible sources included review articles, original research studies, mechanistic studies, human studies, translational animal studies, clinical studies, pharmacological studies, and drug development papers.

Articles were excluded if they were non-English, duplicates, inaccessible as full text, unrelated to appetite regulation or energy balance, focused only on non-peptide pathways, or addressed general nutrition, cardiovascular disease, diabetes, or inflammation without a clear connection to appetite-regulating peptides or gut–brain signalling. Animal studies were excluded if their relevance to human appetite regulation or translational mechanisms was limited.

### 2.4. Selection of Sources of Evidence

Records were screened first by title and abstract. Articles that appeared relevant to peptide-mediated appetite regulation or therapeutic modulation were then assessed in full text. Selection was based on relevance to the review aim, mechanistic contribution, and coverage of central pathways, peripheral peptide signalling, gut–brain communication, microbiota-related pathways, appetite dysregulation, or pharmacological modulation.

### 2.5. Data Charting

Data from included sources were charted narratively using predefined categories: peptide or hormone studied, source tissue, receptor or signalling pathway, role in hunger or satiety, central or peripheral site of action, disease relevance, therapeutic implication, and limitations of the evidence. Findings were then grouped into thematic sections covering peptide structure and signalling, central hypothalamic regulation, peripheral peptide hormones, gut–brain axis signalling, microbiota-related modulation, appetite network dysregulation, and therapeutic strategies.

### 2.6. Synthesis of Results

Because this was a scoping review, findings were synthesised narratively rather than statistically. Evidence was organised to map how peptide hormones contribute to appetite regulation across molecular, cellular, organ-system, and therapeutic levels. Attention was given to pathway overlap, compensatory mechanisms, translational uncertainty, and emerging peptide-based therapies.

### 2.7. Critical Appraisal

A formal risk of bias assessment was not performed, as the objective of this scoping review was to map the breadth and nature of available evidence rather than to evaluate the effectiveness of a single intervention. However, interpretation of the included evidence considered study type, recency, mechanistic relevance, and translational applicability.

### 2.8. PRISMA-ScR Flow Summary

**Figure 1:**
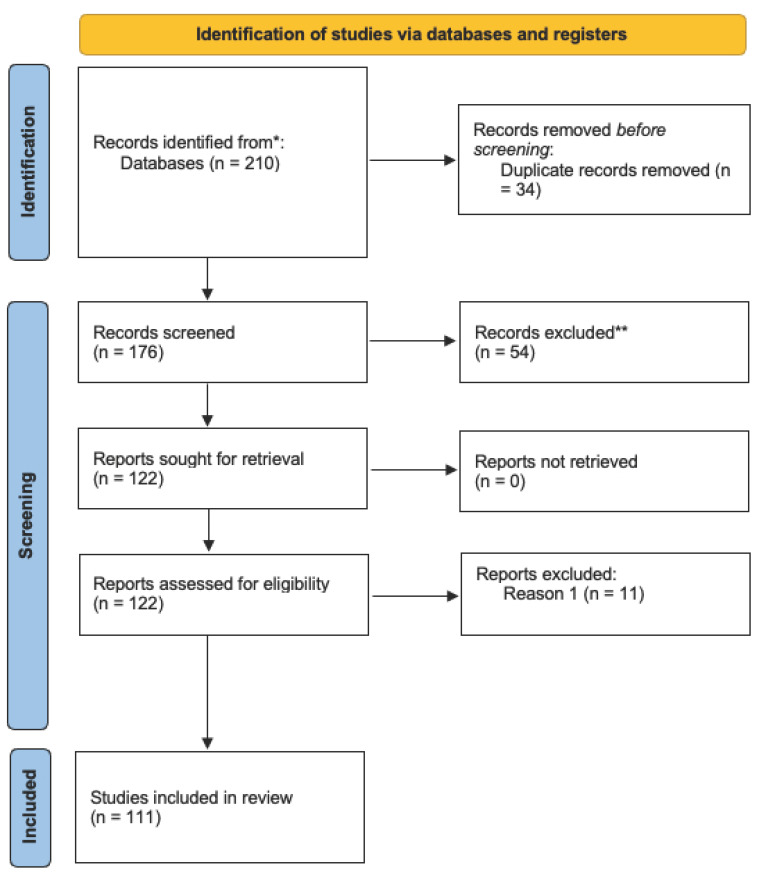
PRISMA 2020 flow diagram for new systematic reviews which included searches of databases and registers only. * Consider, if feasible to do so, reporting the number of records identified from each database or register searched (rather than the total number across all databases/registers). ** If automation tools were used, indicate how many records were excluded by a human and how many were excluded by automation tools.

A total of approximately 210 records were identified through database searching and manual reference list screening. After removing 34 duplicates, 176 records were screened by title and abstract. Of these, 54 records were excluded because they were not directly relevant to appetite regulation, peptide hormones, obesity, or gut–brain signalling. A total of 122 full-text articles were assessed for eligibility, and 11 articles were excluded due to limited relevance, inaccessible full text, or insufficient focus on peptide-mediated appetite regulation. Overall, 111 articles were included in the final review (Figure 1).

## 3. Central and Peripheral Regulation of Appetite

Appetite regulation is governed by a continuous communication between peripheral hormonal signals and central neural circuits, forming an integrated network that maintains energy homeostasis [19]. The central nervous system (CNS) interprets signals from peripheral organs such as GI tract, pancreas and adipose tissue, and initiates responses accordingly to coordinate feeding behaviour, energy expenditure, and metabolism. This complex network maintains short-term appetite control including hunger and satiety, as well as long-term energy balance and body weight stability [20].

### 3.1. Central Regulation of Appetite: Hypothalamic Peptide Networks

The hypothalamus serves as the primary centre for the regulation of appetite and energy homeostasis [21]. Within this region, the ARC, located adjacent to the third ventricle and median eminence, acts as a critical hub for appetite control due to its ability to sense fluctuations in circulating nutrients and hormones [17]. The ARC contains two key neuronal populations with opposing effects on feeding behaviour: NPY/AgRP neurons, which stimulate food intake, and anorexigenic POMC/CART neurons, which suppress appetite [22].

Orexigenic neuropeptides, including NPY and AgRP, are activated during states of negative energy balance to promote food intake. NPY, a potent 36-amino-acid peptide neurotransmitter of the pancreatic polypeptide family (YPSKPDNPGEDAPAEDLARYYSALRHYINLITRQRY-NH_2_), stimulates appetite through actions in the hypothalamus [23,24]. Structurally, it adopts a conserved PP-fold, comprising an *N*-terminal polyproline helix and a *C*-terminal α-helix connected by a hairpin turn, with an amidated *C*-terminus essential for binding to GPCRs and mediating its biological effects [25]. AgRP, coexpressed with NPY, enhances orexigenic signalling by antagonising melanocortin-4 receptors (MC4R), thereby inhibiting the satiety effects of α-MSH [26,27]. While genetic deletion of AgRP produces only mild phenotypic effects, central administration induces a sustained increase in food intake, suggesting a key role in maintaining prolonged feeding rather than initiating acute hunger [28]. In addition to NPY and AgRP, the inhibitory neurotransmitter γ-aminobutyric acid (GABA) is also released from orexigenic ARC neurons, where it suppresses anorexigenic POMC neuronal activity to further promote feeding behaviour [29].

In contrast, anorexigenic neurons, particularly POMC and CART neurons, are activated under conditions of energy sufficiency and in response to signals such as leptin and insulin [17]. POMC is cleaved to produce α-MSH, which promotes satiety and increases energy expenditure via activation of MC4R [23]. CART signalling acts synergistically with POMC pathways to further suppress feeding and stabilise energy intake when physiological requirements are met [26].

Additional hypothalamic regions contribute to the regulation of appetite and energy homeostasis. The paraventricular nucleus (PVN) integrates inputs from both orexigenic and anorexigenic neurons of the ARC and releases neuropeptides such as CRH and TRH, which suppress food intake and regulate autonomic and neuroendocrine responses [30,31]. The lateral hypothalamus (LH) functions as a key feeding centre, promoting appetite and feeding behaviour, partly through orexin and melanin-concentrating hormone (MCH) signalling [20,32,33,34]. In contrast, the ventromedial nucleus (VMN) is primarily associated with satiety, being activated by anorexigenic signals to inhibit food intake [30].

### 3.2. Peripheral Regulation of Appetite: Hormonal and Neural Peptide Signalling

Peripheral signals regulating appetite originate primarily from the GI, pancreas, and adipose tissue, providing information about nutrient status and energy reserves [26]. These signals are conveyed to the central nervous system via the bloodstream and vagal afferent pathways, where they modulate hypothalamic and brainstem circuits involved in appetite control [20,35].

**Figure 2:**
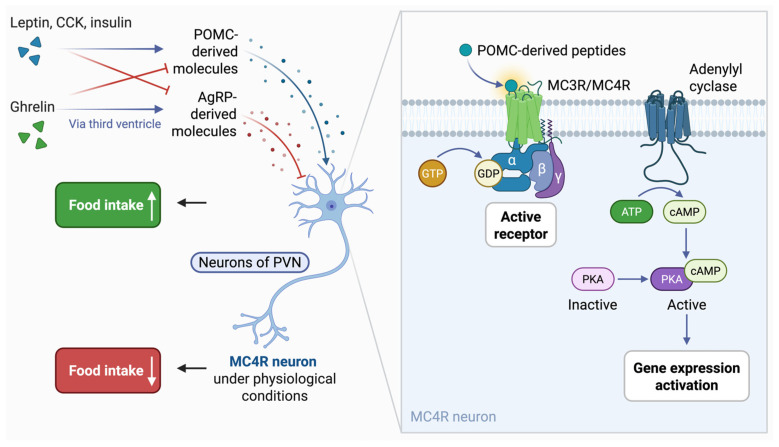
AgRP neurons: Leptin-driven regulation of hunger and satiety via POMC/CART activation. AgRP, agouti-related peptide; cAMP, cyclic adenosine monophosphate; CART, cocaine- and amphetamine-regulated transcript; CCK, cholecystokinin; GDP, guanosine diphosphate; GTP, guanosine triphosphate; MC3R, melanocortin-3 receptor; MC4R, melanocortin-4 receptor; PKA, protein kinase A; POMC, particularly pro-opiomelanocortin neurons. Red, inhibition; black or navy, activation; white, directions.

Ghrelin, the principal orexigenic peripheral hormone, is secreted by the stomach during fasting and acts on NPY/AgRP neurons in the hypothalamus to stimulate appetite [20]. It is a 28-amino-acid peptide derived from the precursor preproghrelin (117 amino acids), with the mature acylated sequence (GSS(n-octanoyl)FLSPEHQRVQQRKESKKPPAKLQPR) [36]. Preproghrelin is first cleaved to form proghrelin and is subsequently processed into its biologically active form. Biological activity requires acylation by the enzyme ghrelin O-acyltransferase (GOAT), enabling ghrelin to cross the blood–brain barrier (BBB) and bind to growth hormone secretagogue receptors (GHSR) on NPY/AgRP neurons [37,38,39]. This interaction promotes hunger, increases food intake, and influences adiposity and glucose metabolism [23,39]. Circulating ghrelin levels typically rise before meals and decline following food intake [40]. In contrast, anorexigenic gut hormones such as GLP-1 and PYY are released postprandially and act to suppress appetite by inhibiting orexigenic pathways and promoting satiety (Figure 2) [39].

In contrast, anorexigenic peripheral peptides are released postprandially and suppress appetite by transmitting satiety signals to the central nervous system [26]. These include leptin, insulin, GLP-1, PYY, cholecystokinin (CCK), oxyntomodulin, and amylin. As summarised in Table 1, these peptides vary in their tissue of origin, receptor specificity, and mechanisms of action. Leptin and insulin primarily provide long-term signals reflecting energy stores, whereas gut-derived hormones predominantly regulate short-term satiety and meal termination [20]. Despite their diverse sources, these peptides converge on hypothalamic and brainstem circuits to inhibit feeding behaviour and maintain energy homeostasis [41].

### 3.3. Integration of Central and Peripheral Signals

Although central and peripheral aspects of appetite regulation are often discussed separately, feeding behaviour arises from their continuous interaction within integrated hypothalamic circuits. Peripheral peptide hormones do not act as isolated on and off switches for hunger or satiety. Instead, they adjust the activity of central neurons, particularly within the ARC, allowing the brain to adapt appetite and energy expenditure to changing metabolic demands [52].

Signals reflecting energy stores and nutrient availability, such as leptin and insulin, reduce the activity of orexigenic AgRP/NPY while stimulating anorexigenic POMC/CART neurons, reinforcing satiety and supporting energy expenditure [53]. In contrast, ghrelin, which rises during fasting, selectively activates AgRP/NPY and shifts hypothalamic output toward hunger promotion. After food intake, gut-derived peptides including GLP-1, PYY, and CCK suppress orexigenic signalling and strengthen satiety through direct hypothalamic effects and indirect relay via the brainstem [39].

**Figure 3:**
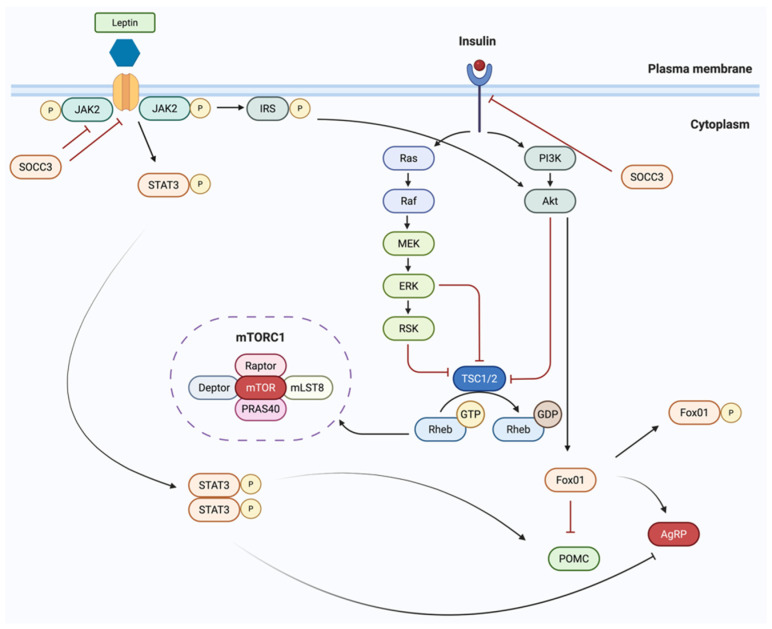
Insulin/leptin intercellular pathway in hypothalamus. AKT, protein kinase B; ERK, extracellular signal-regulated kinase; FoxO1, forkhead box protein O1; JAK2, janus kinase 2; PI3K, phosphoinositide 3-kinase; MEK, mitogen-activated protein kinase kinase; POMC, particularly pro-opiomelanocortin neurons; RAF, rapidly accelerated fibrosarcoma; RAS, rat sarcoma; Rheb, Ras homolog enriched in brain; RSK, ribosomal S6 kinase; STAT3 (signal transducer and activator of transcription 3; TSC, tuberous sclerosis complex; TSC1, hamartin; TSC2, tuberin. Red, inhibition, balk, activation.

Leptin and insulin reshape ARC neuron activity via primary intracellular pathways (leptin: JAK2–STAT3; insulin: PI3K–Akt and MAPK), influencing gene expression, excitability, and synaptic plasticity [54,55]. Disruption of these pathways contributes to central leptin/insulin resistance in obesity, weakening satiety signalling despite high circulating hormone levels (Figure 3) [52,56].

Signals processed within the ARC are distributed across a network of secondary hypothalamic nuclei. Projections to the PVN influence autonomic and neuroendocrine outputs through peptides such as CRH and TRH, linking appetite regulation with stress responses and energy expenditure [23]. Connections to the LH integrate metabolic state with motivational and reward driven aspects of feeding, while inputs to VMN contribute to longer-term regulation of energy expenditure and glucose sensing rather than immediate food intake. Together, these pathways form a distributed circuit that balances hunger, satiety, and metabolic demands [57].

Earlier models of appetite regulation described hypothalamic control as a simple balance between orexigenic and anorexigenic pathways centred on the ARC [16]. While this framework has been instrumental in identifying key peptides and receptors, accumulating evidence indicates that appetite regulation is considerably more complex [52,58,59]. Recent studies suggest that the ARC functions as a heterogeneous network of specialised neuronal subtypes rather than a binary system, with feeding behaviour shaped by context-dependent and overlapping microcircuits [60].

Beyond the diversity observed within the ARC itself, appetite regulation also involves neighbouring hypothalamic and sub hypothalamic regions. Neuronal populations such as inhibitory somatostatin-expressing cells in the tubular nucleus, GABAergic neurons in the zona incerta, and temperature-sensitive neurons of the medial preoptic area contribute to the regulation of feeding behaviour [61]. By integrating metabolic signals with sensory and environmental information, these circuits emphasise that appetite control is distributed across multiple regions and adapts dynamically to physiological context rather than being controlled by a single, central pathway [62].

In a study by Chern and colleagues, NPY was identified as the critical mediator of the sustained effects of AgRP neuron activation on feeding [51]. Selective disruption of AgRP neuron signalling revealed that deletion of NPY, but not AgRP or GABA, abolished optogenetically evoked feeding, an effect that was fully rescued by reinstating NPY expression specifically in AgRP neurons [51]. These findings demonstrate that NPY is uniquely required to maintain hunger during the interval between food seeking and consumption [51]. Complementing this, Krashes and colleagues demonstrated that acute DREADD-mediated activation of AgRP neurons engages distinct neurotransmitters across time: GABA or NPY drives rapid feeding, whereas AgRP, acting via MC4 receptors, mediates delayed but prolonged food intake [29]. Together, these findings delineate temporally distinct neurochemical mechanisms within AgRP neurons, with NPY uniquely sustaining hunger and AgRP contributing to longer lasting orexigenic effects [29].

Understanding how peripheral peptide hormones signal to hypothalamic and brainstem circuits is crucial for designing peptide therapies. It shows whether appetite can be controlled through peripheral receptors alone or if lasting effects require engaging central networks, key parts of the gut–brain axis, alongside vagal signalling, hormonal feedback, and microbiota influences.

## 4. Gut–Brain Axis and Signalling Pathways in Appetite Regulation

The gut–brain axis is a communication system which connects GI tract, peripheral metabolic organs to the brainstem, and CNS. This complex network enables rapid, meal-related regulation of food intake while also contributing to longer-term energy balance. Peptide hormones play a major role within this axis by transmitting information regarding nutrient availability, gut distension, and metabolic state to the CNS [63].

### 4.1. Vagal Afferent Signalling and Brainstem Integration

One of the most rapid routes of gut–brain communication is via vagal afferent fibres, which carry the sensory information from the GI tract to the nucleus of the tractus solitarius (NTS) in the brainstem [64]. Enteroendocrine cells release peptides such as CCK, GLP-1 and PYY in response to nutrient ingestion, which activate receptors on vagal afferent terminals innervating the gut wall [23]. These neural signals reach the NTS within minutes, enabling fast modulation of meal size and termination [23].

The NTS acts as a critical integration hub, where it processes vagal input and relays signals to hypothalamic centres, including the ARC and PVN. This communication allows peripheral signals to influence central appetite circuits without the need for all hormones to cross the BBB. In contrast to endocrine signalling, which operates over longer timescales, vagal afferent pathways provide a rapid feedback mechanism that regulates feeding behaviour during ongoing meals [65].

Non-neuronal cells also actively regulate brain nutrient sensing. At the BBB and circumventricular organs (CVOs) such as median eminence (ME) and area postrema (AP) [66], glial cells control nutrient access by modulating cerebral blood flow, enhanced by pericyte and astrocyte signalling while reduced flow limits ghrelin’s orexigenic effects [67]. They further drive structural remodelling; vascular endothelial growth factor (VEGF) signalling increases capillary permeability during fasting, and oligodendrogenesis in the ME remodels the extracellular matrix to enhance nutrient sensing and neuroendocrine responses [68].

### 4.2. Hormonal Feedback Loops in Short and Long-Term Appetite Control

In parallel with neural pathways, circulating peptide hormones form endocrine feedback loops that regulate appetite across different timeframes. Short-term satiety is primarily regulated by gut-derived peptides such as GLP-1, PYY, and CCK, which rise postprandially and suppress appetite by acting on both brainstem and hypothalamic targets [69]. These hormones reduce meal size, slow gastric emptying, and reinforce sensations of fullness, contributing to the termination of food intake [69].

Long-term energy balance is regulated mostly by adipose- and pancreas-derived hormones such as leptin and insulin, which carry information about overall energy stores rather than individual meals. These hormones modulate hypothalamic circuits by altering the sensitivity of orexigenic and anorexigenic neurons, therefore adjusting baseline appetite and energy expenditure [52].

Disruption of these hormonal feedback loops is a hallmark of metabolic disease. In obesity, for example, leptin and insulin resistance in the hypothalamus reduces anorexigenic signalling, and postprandial gut hormone responses may be reduced [64]. As a result, peripheral signals fail to trigger appropriate central responses, contributing to persistent overeating despite adequate or excessive energy stores [52,64].

### 4.3. Gut Microbiota and Peptide Hormone Signalling

Recent evidence highlights that the gut microbiota adds another layer of control within the gut–brain axis. It can influence appetite indirectly by changing how nutrients are processed, and more directly by affecting the release and activity of gut peptide hormones [59,70]. When gut microbes ferment dietary fibre, they produce short-chain fatty acids (SCFAs). These SCFAs can stimulate enteroendocrine cells to release GLP-1 and PYY, boosting satiety signalling [70].

Alterations in gut microbial composition have been associated with changes in peptide hormone release and the control of appetite. In obesity and type 2 diabetes, studies show reduced abundance of beneficial taxa such as Akkermansia muciniphila and Bifidobacterium, alongside shifts in the Firmicutes to Bacteroidetes ratio [71]. These changes have been linked to lower postprandial GLP-1 and PYY release and weaker satiety signalling. On the other hand, dysbiosis can promote slight systemic inflammation through microbial products such as lipopolysaccharide, which can interfere with hypothalamic appetite regulation and disturbed gut–brain communications [72,73].

**Figure 4:**
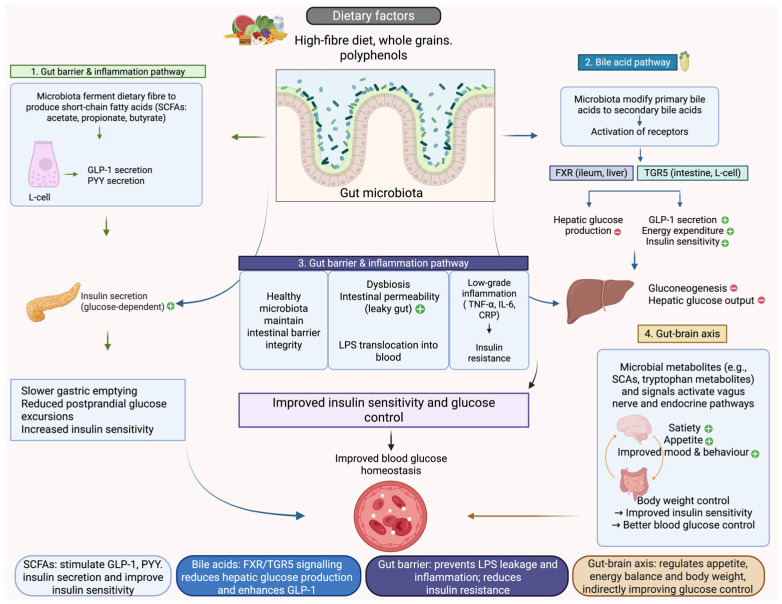
Regulation of blood glucose by gut microbiota. Gut microbiota influence glucose regulation through several interconnected pathways. Fermentation of dietary fibre produces short-chain fatty acids, which stimulate enteroendocrine hormone secretion, including glucagon like receptor-1 (GLP-1) and peptide YY (PYY), supporting insulin secretion and satiety [75]. Microbial modification of bile acids activates farnesoid X receptor (FXR) and takeda G protein-coupled receptor 5 (TGR5) signalling, affecting GLP-1 release, hepatic glucose production and insulin sensitivity [76]. A balanced microbiota also maintains intestinal barrier integrity, reducing lipopolysaccharides (LPS) translocation, systemic inflammation and insulin resistance. In addition, microbial signals communicate with the central nervous system through the gut–brain axis, influencing appetite, body weight and energy balance. Together, these mechanisms contribute to improved blood glucose homeostasis [77]. CRP, C reactive protein; IL-6, L′Interleuchina-6; SCFAs, short-chain fatty acids; TNF-α, tumour necrosis factor alpha.

Overall, the microbiome appears to shape gut–brain signalling in ways that could be therapeutically useful, although many of the underlying mechanisms remain uncertain (Figure 4) [4,74].

### 4.4. Implications for Therapeutic Targeting

Understanding gut–brain signalling has been key to developing today’s peptide therapies for obesity and metabolic disease [78]. Drugs such as GLP-1 receptor agonists build on the body’s own satiety system by boosting gut–brain hormone signalling, which reduces appetite and supports sustained weight loss [78]. However, because of variable clinical response to these therapies, the response of these treatments depends on the hormonal and neural parts of this network being functioning and well integrated.

## 5. Therapeutic Modulation of Appetite-Regulating Pathways

A better understanding of peptide-mediated appetite regulation has reshaped therapeutic approaches to obesity and metabolic disease, shifting emphasis from appetite suppression alone toward targeted modulation of gut–brain signalling, hypothalamic integration, reward-related feeding, and metabolic control [79]. Because appetite regulation is redundant and adaptive, therapeutic strategies are increasingly being considered according to their physiological outcomes, including satiety induction, suppression of orexigenic drive, reward modulation, and coordinated multi-hormone signalling.

### 5.1. Satiety Induction and Postprandial Signal Amplification

**Figure 5:**
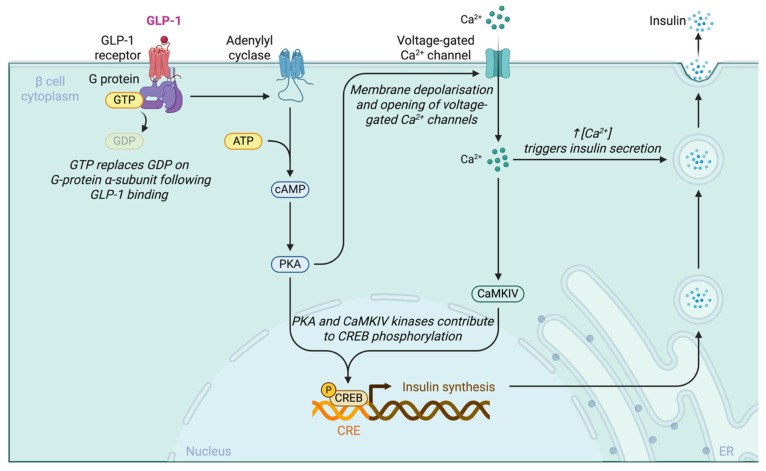
Molecular mechanism of action of glucagon-like peptide-1. cAMP, cyclic adenosine monophosphate; CaMKIV, calcium/calmodulin-dependent protein kinase type IV; CREB, cAMP response element-binding protein; GDP, guanosine diphosphate; GTP, guanosine triphosphate; PKA, protein kinase A.

GLP-1 receptor agonists, including liraglutide and semaglutide, are among the most established peptide-based therapies for obesity and type 2 diabetes [80,81,82]. They reduce food intake through peripheral and central mechanisms, including delayed gastric emptying, vagal afferent signalling, and modulation of hypothalamic and reward-related circuits involved in appetite and food motivation [83]. In type 2 diabetes, GLP-1 receptor activation also improves glycaemic control by enhancing glucose-dependent insulin secretion and suppressing glucagon release, with a low risk of hypoglycaemia (Figure 5) [84].

Dual incretin agonists such as tirzepatide extend this strategy by activating both GLP-1 and GIP receptors, engaging multiple metabolic and satiety pathways simultaneously [85,86]. In the SURMOUNT-5 trial, tirzepatide produced greater weight loss than semaglutide and was associated with larger improvements in health-related quality of life, particularly physical functioning [87]. These findings suggest that multi-pathway incretin activation may partly overcome compensatory mechanisms that limit GLP-1 receptor agonist monotherapy.

Amylin-based therapies provide another route to satiety enhancement. Amylin analogues such as pramlintide and the longer acting cagrilintide reduce appetite by strengthening central satiety signalling and slowing gastric emptying [88,89]. Although their weight-loss effect as monotherapy is usually modest, combining amylin-based signalling with GLP-1 receptor agonism appears to produce stronger metabolic effects. In people with type 2 diabetes, CagriSema (cagrilintide plus semaglutide) improved glycaemic control and produced significantly greater weight loss than either semaglutide or cagrilintide alone. However, HbA1c reduction was greater than cagrilintide alone but not superior to semaglutide, suggesting that the main added value of the combination may be enhanced weight loss rather than additional glucose lowering [90]. These findings support a multi-hormone strategy that more closely resembles normal postprandial peptide signalling and may help overcome some limitations of single-pathway therapy.

### 5.2. Suppression of Orexigenic Drive and Circuit Plasticity

Suppressing hunger promoting pathways has been more challenging than enhancing satiety. Ghrelin is an attractive target because it is the major circulating orexigenic hormone, yet ghrelin-based interventions have shown limited clinical success. Ghrelin receptor antagonists, including [D-Lys3]-GHRP-6, JMV2959, and PF-05190457, have shown appetite-suppressing effects in preclinical or early clinical settings [91,92,93]. Anti-ghrelin vaccination strategies such as CYT009-GhrQb have also been explored [94]. However, sustained weight loss has been difficult to achieve, probably because blocking one orexigenic signal allows parallel appetite pathways to compensate.

Circuit-level evidence helps explain this limitation. Grzelka et al. showed that fasting activates a PVH TRH/PACAP neuronal subset projecting to AgRP neurons, producing long-lasting synaptic amplification during sustained energy deficit [95]. Similarly, AgRP-(83–132) induces prolonged activation across hypothalamic and reward-related regions, including the nucleus accumbens shell, central amygdala, nucleus of the solitary tract, and lateral hypothalamus, whereas NPY responses are shorter-lived [96]. These findings suggest that persistent hunger is maintained not only by peripheral hormones but also by plasticity within orexigenic circuits. Durable appetite suppression may therefore require strategies that address AgRP-related circuit adaptation rather than targeting ghrelin alone [95,96,97].

Melanocortin pathway modulation provides a more downstream central strategy, particularly where appetite dysregulation arises from impaired POMC–α-MSH–MC4R signalling. The MC4R agonist setmelanotide illustrates this approach in rare genetic obesity syndromes involving the melanocortin pathway, where restoring downstream satiety signalling can reduce hyperphagia and body weight [98].

### 5.3. Modulation of Reward-Driven Feeding

Appetite regulation is also shaped by reward pathways that can drive food intake independently of metabolic need. Zhu and colleagues identified a peri-locus coeruleus to ventral tegmental area pathway involved in hedonic feeding, showing that VTA dopamine neurons encode food palatability and bidirectionally control hedonic intake [99]. Semaglutide suppressed VTA dopamine activity during feeding, but this effect weakened with repeated treatment as palatable food intake and dopamine activity recovered. These findings suggest that reward circuits may adapt to GLP-1 receptor agonism and partly oppose homeostatic satiety signalling. Therapeutic strategies that combine gut–brain satiety enhancement with reward-pathway modulation may therefore improve long-term appetite control [99].

### 5.4. Multi-Target Therapies and Energy-Expenditure Pathways

Emerging therapeutic strategies increasingly aim to engage several peptide pathways simultaneously rather than intensifying a single satiety signal. This approach is particularly relevant because appetite regulation is biologically redundant, and single-pathway therapies may be limited by compensatory changes in orexigenic, satiety, and reward-related circuits [95,96,97,99]. Triple agonists targeting GLP-1, GIP, and glucagon receptors extend this principle by combining appetite reduction and glycaemic effects with potential effects on lipid metabolism and energy expenditure. Retatrutide, a GLP-1/GIP/glucagon receptor agonist, produced substantial body-weight reductions in adults with obesity in phase 2 clinical testing [100]. This reflects a broader shift from suppressing food intake alone toward coordinated modulation of both energy intake and energy utilisation. However, these approaches require further evaluation of long-term safety, tolerability, durability after discontinuation, and effects on lean mass [101,102,103,104,105].

### 5.5. Summary of Peptide-Based Therapeutic Strategies

The therapeutic approaches discussed in this review target distinct components of the appetite-regulating network, including satiety pathways, orexigenic circuits, and multi-receptor systems. Table 2 summarises the main peptide-based strategies, their mechanisms of action, and their key limitations.

### 5.6. Translational Challenges

Despite substantial therapeutic progress, peptide-based appetite modulation faces important durability and safety challenges because appetite regulation is biologically adaptive. GLP-1-based therapies can partly bypass impaired endogenous satiety signalling, but central compensatory responses may re-emerge over time, particularly in individuals with leptin or insulin resistance [101]. Clinical studies also show substantial weight regain after treatment discontinuation, suggesting that pharmacological appetite suppression does not permanently reset the underlying biological drivers of energy balance [102,103]. In addition, body-composition analyses indicate that incretin-associated weight loss may include a meaningful reduction in lean mass, raising concerns about muscle preservation, metabolic health, and physical function, especially in older adults or those at risk of sarcopenia [104]. Safety and tolerability also remain key translational barriers: gastrointestinal adverse effects are common and may contribute to discontinuation, while less frequent concerns such as gallbladder disease and delayed gastric emptying highlight the need for careful patient selection, dose escalation, and long-term pharmacovigilance [105].

## 6. Future Perspectives

Incretin-based therapies have transformed obesity care, but their limits are pushing research toward new targets beyond the classic appetite hormones. Increasingly, obesity is understood as a system-level disorder involving interacting changes in genetic regulation, metabolic signalling, neural circuitry, and gut microbiota composition. This has fuelled interest in multi-pathway strategies and newer targets, such as the GDF15–GFRAL axis, bile acid receptors (FXR and TGR5), and adipose-derived signalling molecules, that may influence appetite, energy expenditure, and metabolic efficiency through different routes [106].

Alongside this, rational combination therapies; for example, pairing incretin agonists with Amylin analogues or other metabolic modulators, are being developed to better match the body’s natural redundancy and reduce the compensatory adaptations that can limit monotherapy. Looking ahead, advances in multi-omics profiling and drug delivery technologies may support more personalised treatment, tailoring therapy to an individual’s neuroendocrine and metabolic phenotype and improving long-term, patient-centred outcomes [107,108].

## 7. Conclusions

Appetite regulation is mostly understood as a complex network rather than a simple on and off switch. Peptide hormones from the gut, adipose tissue and pancreas connect and converge in the hypothalamus and reward circuit, which integrates internal energy needs with external factors such as environment, stress and eating behaviours. Dysregulation of this system can come from impaired hormonal signalling, altered neuronal sensitivity, or dominance of reward pathways, which can contribute to conditions such as obesity. Understanding of this system has enabled better peptide-based therapies for obesity and type 2 diabetes, especially incretin-based drugs and newer multi-agonist approaches. However, biological redundancy, hormone resistance, and individual differences mean that the treatments are not universally effective. Effective interventions should target gut–brain communications and support vagal signalling and gut microbiota stability, reinforcing the stability of the neural circuits that interpret metabolic cues, and also focus on the therapies that can shift the mindset around food from pleasure-based consumption to just seeing it as a fuel for the body.

## Figures and Tables

**Table 1:** Anorexigenic peptide hormones in appetite regulation [20,35,42,43].

Anorexigenic Peptides (Appetite-Suppressing)	Primary Source	Target	Central Pathway	Mechanism/Effect on Appetite	Clinical Relevance	Ref
Leptin (167-mer)	Adipose tissue	LepR	Activates POMC/CART neurons; inhibits NPY/AgRP neurons in ARC	Reduce food intake and increase energy expenditure	Chronic overnutrition causes leptin resistance in obesity, blunting its anorexigenic effect	[44,45]
Insulin (51-mer)	Pancreatic β-cells	Insulin Receptor	Activating hypothalamic ARC neurons. Inhibits NPY/AgRP and stimulates POMC neurons	Circulating insulin crosses BBB, Decreasing appetite, Regulate glucose	Impaired central insulin signalling in obesity and T2DM	[46]
Peptide YY (36-mer)	Ileum & colon L cells	Y2 receptor	Inhibits NPY neurons in ARC	Suppress appetite post-prandially	Reduced response observed in obesity	[47]
GLP-1 (7-36) (31-mer)	Small intestine (L-cells)	GLP 1 receptor	Acts via nucleus of the tractus solitarius (NTS) and hypothalamus; slows gastric emptying	Secreted after nutrient intake; slows gastric emptying, enhances insulin secretion (incretin effect) and suppress appetite	GLP-1 receptor agonists (e.g., semaglutide) used in obesity and diabetes	[48]
CCK (8-mer)	Duodenum and Jejunum	CCK-A receptor	Vagal afferent → NTS → hypothalamus	Early meal termination, Reduces meal size	Short-term satiety signal	[49]
Oxyntomodulin (OXM), (37-mer)	Intestinal L-cells	GLP-1 & glucagon receptors	Hypothalamic satiety pathway	Decreases food intake and may enhance energy expenditure.	Dual-agonist potential	[50]
Amylin (37-mer)	Pancreatic β-cells	Calcitonin receptor	Acts on brainstem and hypothalamus	Cosecreted with insulin; slows gastric emptying and promotes satiety via central amylin receptors	Amylin analogues (pramlintide) used clinically	[51]

AgRP, agouti-related peptide; cAMP, cyclic adenosine monophosphate; CART, cocaine- and amphetamine-regulated transcript; CCK, cholecystokinin; GLP-1, glucagon like receptor; GDP, guanosine diphosphate; GTP, guanosine triphosphate; LepR, leptin receptor; MC3R, melanocortin-3 receptor; MC4R, melanocortin-4 receptor; NPY, neuropeptide Y; NTS, nucleus of the tractus solitarius; PKA, protein kinase A; POMC, particularly pro-opiomelanocortin neurons, Y2 receptor, neuropeptide Y receptor Y2.

**Table 2:** Peptide-based therapies targeting appetite regulation [83,85,88,89,94,100].

Therapeutic Outcome	Therapy Class	Example Agents	Primary Mechanism	Clinical Benefit	Key Limitations
Satiety induction	GLP-1 receptor agonists	Liraglutide, semaglutide	Delayed gastric emptying; central satiety signalling	Weight loss; improved glycaemic control; cardiovascular benefit	GI side effects; variable response
Multi-pathway satiety/metabolic control	Dual incretin agonists	Tirzepatide	GLP-1 + GIP receptor activation	Greater weight loss and QoL improvement than GLP-1 monotherapy	Long-term safety data still developing
Satiety enhancement	Amylin analogues	Pramlintide, cagrilintide	Enhanced satiety; slowed gastric emptying	Reduced meal size; additive benefit with GLP-1 therapy	Limited efficacy as monotherapy
Orexigenic pathway suppression	Ghrelin antagonists/vaccines	[D-Lys3]-GHRP-6, JMV2959, PF-05190457, CYT009-GhrQb	Suppression of ghrelin/GHSR signalling	Theoretical appetite reduction	Circuit compensation; limited durable efficacy
Central satiety restoration	MC4R agonists	Setmelanotide	Activates melanocortin signalling downstream of POMC/α-MSH	Reduced hyperphagia in selected genetic obesity syndromes	Limited to defined pathway defects
Integrated gut-hormone modulation	Combination or multi-agonist therapies	GLP-1 + amylin; GLP-1/GIP/glucagon agonists	Mimic multiple postprandial and metabolic signals	Improved efficacy potential	Cost, tolerability, treatment complexity

## Data Availability

No new data were generated or analysed in this study. All information presented is based on previously published literature.
